# The screening and identification of DNA barcode sequences for *Rehmannia*

**DOI:** 10.1038/s41598-019-53752-8

**Published:** 2019-11-21

**Authors:** Hongying Duan, Wanshen Wang, Yunpeng Zeng, Mengmeng Guo, Yanqing Zhou

**Affiliations:** College of Life Sciences, Henan Normal University, Xinxiang, 453007 Henan China

**Keywords:** PCR-based techniques, Genetic markers, Plant molecular biology

## Abstract

In this study, ITS, ITS2, *matK*, *rbcL* and *psbA-trnH* in *Rehmannia* were successfully amplified and sequenced, but some ITS sequences need to be proofread according to ITS2 sequences. Compared with *rbcL*, *matK* and *psbA-trnH*, ITS and ITS2 had higher mutation rate and more information sites, and ITS2 had higher interspecific diversity and lower intraspecific variation in *Rehmannia*, but the interspecific genetic variation of *rbcL* and *matK* was lower. Furthermore, the obvious barcoding gap was found in *psbA-trnH* or ITS2 + *psbA-trnH*, and the overlap between interspecific and intraspecific variation of ITS, ITS2 or *matK* was less. In addition, the phylogenetic tree based on ITS or ITS2 indicated that *R. glutinosa*, *R. chingii* or *R. henryi* with obvious monophyly could be successfully identified, but *R. piasezkii and R. elata* were clustered into one branch, *R. solanifolia* could not be distinguished from *R. glutinosa*, and *R. chingii* was closer to *R. henryi*. In phylogenetic tree based on *psbA-trnH* or ITS2 + *psbA-trnH*, cultivars and wild varieties of *R. glutinosa* could be distinguished, were clearly separated from other *Rehmannia* species, and cultivars or wild varieties of *R. glutinosa* could be also distinguished by *matK*. Taken together, ITS2 has great potential in systematic study and species identification of *Rehmannia*, the combination of ITS2 and *psb*A-*trn*H might be the most suitable DNA barcode for *Rehmannia* species.

## Introduction

*Rehmannia* is composed of six species such as *R. solanifolia*, *R. chingii*, *R. henryi*, *R. piasezkii*, *R. elata* and *R. glutinosa*, except *R. glutinosa* distributes in East Asia and Japan, other *Rehmannia* species are only distributed in China^1^. *Rehmannia* species have the same medicinal constituents, but *R. glutinosa* possess higher content of medicinal constituents than other five species, such as catalpol, verbascoside and others^2^, thus *R. glutinosa* has important medicinal value, edible value and health care effect, and is widely reported. At present, *R. glutinosa* has been studied in clinic use, medicinal constituent, breeding, cultivation, classification, tissue culture and so on. However, the wild resources of *R. glutinosa* have been excessively exploited, planting area and harvest amount of *R. glutinosa* also decrease. Therefore, effective classification technique based on genetic variation need to be investigated for *Rehmannia* species and the varieties and adulterants of *R. glutinosa*.

DNA barcoding is a rapid and accurate technique for species discrimination with short DNA fragment, is necessary for the authentication of medicine plant^3^, is complementary for traditional identification^4^, DNA barcoding also has clinical, agricultural, forensic, illegal trade-related, ecological and recreational applications^5^. At present, DNA barcoding has been hotspot in biotaxonomy, but there are still debates on which DNA region can be used as the standard barcode for land plants. Some markers in chloroplast genome or plastid DNA regions have been explored as DNA barcodes, such as *matK*, *trnH-psbA*, *rbcL*, *atpF-atphH*, *rpoB*, *psbK-psbI*r and *rpoC1*^6^, and some nuclear ribosome DNA sequences including internal transcribed spacer (ITS), internal transcribed spacer1 (ITS1), internal transcribed spacer 2 (ITS2) and so on also have been evaluated^3^, but each of these sequences does not conform to the principle of DNA barcoding because of some drawbacks, for example, low rate of variation and amplification, poor universality of primer, gene deletion and so on. More and more researchers recommend the integration of DNA barcode to classify and identify species^7^, and many different combinations of DNA barcodes have been put forward for different plants, such as *rbcL* + *psbA-trnH*^8^, *rpoC1* + *matK* + *psbA-trnH*^9^, ITS2 + *psbA-trnH*^10^, ITS + ITS2 + *matK* + *rbcL* + *psbA-trnH*^11^ and so forth.

At present, among these candidate DNA barcodes of plant, ITS is used in analysis of genetic variation between cultivars and varieties of *R. glutinosa*^12^, assessment of systematic relationships in *Rehmannia* species^13^ and study of DNA barcode for identification of *Rehmannia* species^14^. *Rehmanniae* Radix and its closely related species could be identified by ITS2^1^, *rbcL*, *ndhF*, *rps*16 and *trnL-F* are applied to study genetic diversity, population genetic relationship and taxology of *R. glutinosa*^15^. However, each of these DNA barcodes all has a few of shortcomings, integrated DNA barcodes have been used in species identification of *Rehmannia*, for example, identification rate of ITS, *trnL-trnF, trnM-trnV and trnS-trnG* all was less than 20% for five species in *Rehmannia*, while that of the combined DNA barcode *trnS-trnG* + ITS was up to 100%, the same as that of *trnL-trnF* + *trnM-trnV* + *trnS-trnG* + ITS^14^. In this study, five candidate DNA barcodes ITS, ITS2*, matK*, *rbcL* and *psbA-trnH* were evaluated to obtain suitable DNA barcode of *Rehmannia*, which would supply theory basis for screening of DNA barcode and species discrimination in plant, furthermore provide more precise and reliable method to identify herbal medicine from medicinal plant in *Rehmannia*.

## Results

### Efficiency of PCR amplification

It was found that primers of ITS, ITS2 and *psbA-trnH* had good universality, but primers for *matK* and *rbcL* need to be screened and optimized in *Rehmannia*. In this study, the full-length sequence of *rbcL* in *Rehmannia* was amplified by the amplification of two overlapping segments, four pairs of primers 1F-724R, 636F-1368R, 5′F-z895R and z674F-3′R were used, these primers had better amplification effects, especially 1F-724R and 636F-1368R. Furthermore, the full-length sequence of *matK* in *Rehmannia* was amplified by two pairs of primers, trnk3914F-trnk2R and 1F-trnk2R, but their length was different and was respectively 2500 bp or 1800 bp, and the amplification efficiency of primer 1F-trnk2R was higher.

In this study, PCR amplification conditions of target sequences were optimized and established, the procedure of PCR amplification was 30 cycles followed by final extension for 10 min at 72 °C, each cycle was composed of pre-degeneration for 3 min at 94 °C, degeneration for 30 s at 94 °C, annealing for 30 s at suitable temperature, extension for 1 min at 72 °C, the annealing temperature for amplification of ITS, ITS2, *rbcL*, *matK* and *psbA-trnH* was 46 °C, 48 °C, 50 °C, 48.6 °C or 55 °C, respectively, and amplification band of target sequences in *Rehmannia* was single, bright and specific (Figs. S1–S5). Sequencing atlas of most target sequences in *Rehmannia* were clear, the baselines were smooth and neat, but overlapping peaks were found in some sequencing results of ITS, thus multiple amplification and sequencing of ITS in *Rehmannia* were required, and ITS sequence was proofread basing on ITS2 sequence. In addition, sequencing rate and acquisition rate of candidate barcodes all were 100% (Table S1), and BLAST results based on sequence matching were shown in Table S2.

### Analysis of sequence characteristics

In order to investigate sequence of candidate barcodes in a wide range, target sequences amplified in this study (Table 1) were analyzed together with relevant data of *Rehmannia* in GenBank database (Table 2), it was found that lengths of target sequences in *Rehmannia* were different (Table 3), ITS2 was the shortest and composed of 224–235 bp with 64.22–66.67% GC, *matK* was the longest and composed of 1536–1560 bp with 33.27–33.53% GC. ITS was 610–614 bp with 60.20–62.32% GC, *rbcL* was 1287 bp with 43.36–43.75% GC, *psbA-trnH* was 483–497 bp with the lowest GC content of 26.76–27.12%.

**Table 1:** Germplasms of *Rehmannia* used in this study and accession number of candidate barcodes.

No.	Chinese name	Species name	Location	GenBank Acc. No.				
				ITS	ITS2	*matK*	*rbcL*	*psbA-trnH*
1	Shangzuo1	*R. glutinosa*	WIAS	KX361133	KX361133	KX347929	KY441581	KY488689
2	Shangxibeixiang	*R. glutinosa*	WIAS	KX361134	KX361134	KX347930	KY441582	KY488690
3	Jinxiandiaoyu	*R. glutinosa*	WIAS	KX361135	KX361135	KX347931	KY441583	KY488691
4	Mixianyesheng	*R. glutinosa*	WIAS	KX361136	KX361136	KX347932	KY441584	KY488692
5	Guolimao	*R. glutinosa*	WIAS	FJ770223	FJ770223	KX347933	KY441585	KY488693
6	Hongshuwang	*R. glutinosa*	WIAS	KX361137	KX361137	KX347934	KY441586	KY488694
7	9302	*R. glutinosa*	WIAS	EU787018	EU787018	KX347935	KY441587	KY488695
8	Kangyu831	*R. glutinosa*	WIAS	FJ770230	FJ770230	KX347936	KY441612	KY488696
9	Guoxianshouji	*R. glutinosa*	WIAS	KX361138	KX361138	KX347937	KY441588	KY488697
10	Huanghouza	*R. glutinosa*	WIAS	KX361139	KX361139	KX347938	KY441589	KY488698
11	Beijing 3	*R. glutinosa*	WIAS	FJ770244	FJ770244	KX347939	KY441590	KY488699
12	Yesheng	*R. glutinosa*	WIAS	KX361140	KX361140	KX347940	KY441591	KY488700
13	Sankuai	*R. glutinosa*	WIAS	FJ770235	FJ770235	KX347941	KY441592	KY488701
14	Xiuwufangzhuang	*R. glutinosa*	WIAS	KX361141	KX361141	KX347942	KY441613	KY488712
15	Wenhuai	*R. glutinosa*	WIAS	KX361142	KX361142	KX347943	KY441593	KY488702
16	Beijing2	*R. glutinosa*	WIAS	FJ770219	FJ770219	KX347944	KY441594	KY488703
17	Jinzhuangyuan	*R. glutinosa*	WIAS	KX361143	KX361143	KX347945	KY441595	KY488704
18	Fanshandihuang	*R. glutinosa*	WIAS	KX361144	KX361144	KX347946	KY441596	KY488705
19	Shizitou	*R. glutinosa*	WIAS	FJ770243	FJ770243	KX347947	KY441597	KY488706
20	Shangzuo2	*R. glutinosa*	WIAS	KX361145	KX361145	KX347948	KY441598	KY488707
21	Zhangsi961	*R. glutinosa*	WIAS	KX361146	KX361146	KX347949	KY441599	KY488708
22	Zhangsi901	*R. glutinosa*	WIAS	KX361147	KX361147	KX347950	KY441600	KY488709
23	Jinjiu	*R. glutinosa*	WIAS	KX361148	KX361148	KX347951	KY441601	KY488710
24	Dihuang	*R. glutinosa*	JCJSC	KX361149	KX361149	KX347953	KY441602	KY488713
25	Dihuang	*R. glutinosa*	MWCJSC	KX361150	KX361150	KX347954	KY441603	KY488714
26	Dihuang	*R. glutinosa*	MTTCJSC	KX361151	KX361151	KX349706	KY441604	KY488715
27	Dihuang	*R. glutinosa*	HNUXHC	KX361152	KX361152	KX347952	KY441605	KY488711
28	Dihuang	*R. glutinosa*	LCHC	KX348047	KX348047	KX349707	KY441606	KY488716
29	Lieyedihuang	*R. piasezkii*	ECNUSC	KX361157	KX361157	KX349708	KY441611	KY488721
30	Dihuang	*R. glutinosa*	SDLHC	KX361153	KX361153	KX349709	KY441607	KY488717
31	Dihuang	*R. glutinosa*	WHC	KX361154	KX361154	KX349710	KY441608	KY488718
33	Dihuang	*R. glutinosa*	HCHC	KX361155	KX361155	KX349711	KY441609	KY488719
34	Dihuang	*R. glutinosa*	SDXHC	KX361156	KX361156	KX349712	KY441610	KY488720

WIAS: Wenxian institute of agricultural sciences, Henan, China; JCJCSC: Junbu, Changqing district, Jinan, Shandong, China; MWCJSC: Mount Wenchang, Changqing district, Jinan, Shandong, China; MTTCJSC: Mount Tai, Taian County, Jinan, Shandong, China; ECNUSC: East China Normal University, Shanghai, China; HNUXHC: Henan Normal University, Xinxiang, Henan, China; LCHC: Lingbao County, Henan, China; SDLHC: Suburban district, Luohe, Henan, China; WHC: Weihui, Henan, China; HCHC: Hui County, Henan, China; SDXHC: Suburban district, Xinxiang, Henan, China.

**Table 2:** GenBank accession number of related sequences in *Rehmannia*.

Species	GenBank Acc. No			
	ITS	ITS2	*rbcL*	*matK*
*Rehmannia chingii*	DQ069313	DQ069313	EF544598	
	EF363673	EF363673	FJ172724	
*R. solanifolia*	DQ069314	DQ069314	FJ172723	
	EF363672	EF363672		
*R. elata*	DQ069315	DQ069315	HQ384874	HQ384505
*R. piasezkii*	DQ069316	DQ069316	FJ172721	
	EF363670	EF363670		
*R. henryi*	DQ272447	DQ272447	FJ172722	
	EF363671	EF363671		
*R. glutinosa*	DQ069312	DQ069312	FJ172725	
	EF363674	EF363674	AJ247615	
	EU787017	EU787017		
	EU787018	EU787018		
	EU810383	EU810383		
	EU810384	EU810384		
	EU810385	EU810385		
	EU810386	EU810386		
	FJ770218	FJ770218		
	FJ770220	FJ770220		
	FJ770221	FJ770221		
	FJ770222	FJ770222		
	FJ770223	FJ770223		
	FJ770224	FJ770224		
	FJ770225	FJ770225		
	FJ770226	FJ770226		
	FJ770227	FJ770227		
	FJ770228	FJ770228		
	FJ770229	FJ770229		
	FJ770230	FJ770230		
	FJ770231	FJ770231		
	FJ770232	FJ770232		
	FJ770233	FJ770233		
	FJ770234	FJ770234		
	FJ770235	FJ770235		
	FJ770236	FJ770236		
	FJ770237	FJ770237		
	FJ770238	FJ770238		
	FJ770239	FJ770239		
	FJ770240	FJ770240		
	FJ770241	FJ770241		
	FJ770242	FJ770242		
	FJ770243	FJ770243		
	FJ770244	FJ770244		
	FJ770245	FJ770245		
	FJ770246	FJ770246		
	FJ770247	FJ770247		
	FJ770248	FJ770248		
	FJ770249	FJ770249		
	FJ980430	FJ980430		
	KR052187	KR052187		
		GQ434798

**Table 3:** Sequence characteristics of candidate barcodes.

Marker	ITS	ITS2	*rbcL*	*matK*	*psbA-trnH*
Sequence length	610–614	224–235	1287	1536–1560	483–497
Alignment length	610	225	1287	1560	497
GC content(%)	60.20–62.32	64.22–66.67	43.36–43.75	33.27–33.53	26.76–27.12
Conserved sites	544	189	1279	1529	482
Variable sites	66	36	8	7	5
Informative sites	35	18	1	4	2
Aberration rate(%)	10.82	16	0.6	0.4	1

As shown in Table 3, ITS had 544 conserved sites, 66 variable sites and 35 informative sites, and the aberration rate of ITS was 10.82% in *Rehmannia*. ITS2 had 189 conserved sites, 36 variable sites and 18 informative sites, and its aberration rate was up to 16% in *Rehmannia*. Moreover, these variable sites in ITS and ITS2 were mainly base substitutions between purine and purine, or pyrimidine and pyrimidine. Compared with ITS and ITS2 in *Rehmannia*, *rbcL*, *matK* and *psbA-trnH* were relatively conserved with low aberration rate and fewer informative sites, for example, there were only 8 variable sites and 1 informative site in *rbc*L with 1279 conserved sites and 99.98% interspecific similarity of *Rehmannia*, the similarity of *matK* in *Rehmannia* was 98.43%, however sequences of *matK* in cultivars of *R. glutinosa* were obviously different from its wild species.

### Determination of genetic divergence

The interspecific and intraspecific divergence of candidate barcodes in *Rehmannia* were calculated with K2P model (Table 4), three parameters of average interspecific distance, average theta prime (θ′) and minimum interspecific distance were used to characterize interspecific divergence of target sequences in *Rehmannia*, it was found that ITS2 showed the highest interspecific diversity, followed by ITS, and the interspecific diversity of *rbcL* was the lowest. Wilcoxon signed rank tests confirmed that there were significant differences among interspecific variations of different target sequences in *Rehmannia* (Table S3), the interspecific variation of ITS2 was extremely significantly greater than that of other sequences, and the variation degree was ITS2 > ITS > *psbA-trnH* > *matK* > *rbcL*, in turn.

**Table 4:** Intraspecific and interspecific genetic divergences of candidate barcodes.

Marker	ITS	ITS2	*rbcL*	*matK*	*psbA-trnH*
All interspecific distance	0.017 ± 0.005	0.027 ± 0.010	0.001 ± 0.001	0.002 ± 0.001	0.009 ± 0.004
Theta prime	0.019 ± 0.005	0.032 ± 0.011	0.001 ± 0.000	0.002 ± 0.001	0.009 ± 0.004
Minimum interspecific distance	0.017 ± 0.005	0.030 ± 0.010	0.0003 ± 0.000	0.001 ± 0.001	0.006 ± 0.003
All intraspecific distance	0.005 ± 0.002	0.005 ± 0.003	0.000 ± 0.000	0.000 ± 0.000	0.002 ± 0.001
Theta	0.001 ± 0.000	0.001 ± 0.001	0.002 ± 0.001	0.000 ± 0.000	0.002 ± 0.001
Coalescent depth	0.004 ± 0.002	0.008 ± 0.003	0.002 ± 0.001	0.002 ± 0.001	0.004 ± 0.003

In addition, average intraspecific distance, theta (θ) and average coalescent depth can reflect on intraspecific divergence of target sequence, and the intraspecific variation of *rbcL* and *matK* in *Rehmannia* was relatively low (Table 4), Wilcoxon signed rank tests indicated that the intraspecific variation of ITS was significantly greater than that of ITS2, *matK* and *rbcL* (Table S4). Moreover, the intraspecific variation of ITS2, ITS and *psb*A-*trnH* was significantly lower than their interspecific variation, and the variation degree of ITS2 in *Rehmannia* was more significant (Table 4), which is beneficial to accurate identification of *Rehmannia* species. Further analysis showed that the combination of ITS2 and *psbA-trnH* had higher interspecific diversity and lower intraspecific divergence.

### Assessment of barcoding gap

**Figure 1:**
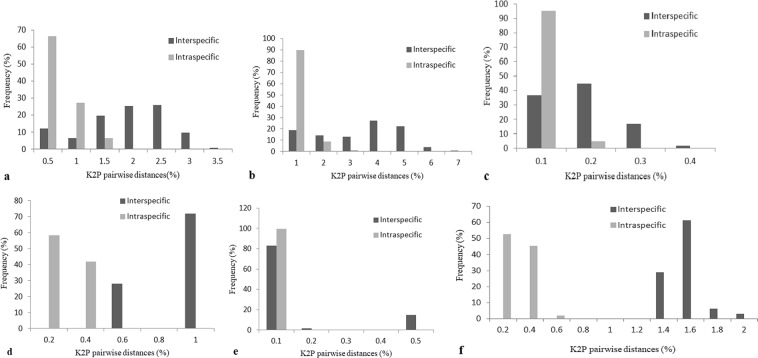
The distribution of K2P pairwise distances for candidate barcodes of *Rehmannia*. (**a**–**f**) respectively represented the distribution of K2P pairwise distance for ITS, ITS2, *matK*, *psbA-trnH*, *rbcL*, or ITS2 + *psbA-trnH* of *Rehmannia*.

The distribution of interspecific and intraspecific variation of target sequences in *Rehmannia* was investigated (Fig. 1), it was found that interspecific and intraspecific variation of *psbA-trnH* did not overlap, there was a significant barcoding gap, the intraspecific variation was concentrated on the left side, and the interspecific variation was concentrated on the right side (Fig. 1d). Furthermore, the distribution of interspecific and intraspecific variation of ITS2 + *psbA-trnH* exhibited obvious barcoding gap, and the gap distance was larger, from 0.6% to 1.4% (Fig. 1f).

Although the obvious barcoding gap was not shown between interspecific and intraspecific variation of ITS, ITS2 or *matK* (Fig. 1a–c), the overlap of genetic variation was less, the distribution of their intraspecific variations mainly concentrated on the left side, and their interspecific variations were mainly distributed on the right side, indicating that their interspecific variations were generally more than intraspecific variations. However, the significant overlap without gap was found in genetic variation of *rbcL* (Fig. 1e).

### Identification effect of candidate barcodes

**Figure 2:**
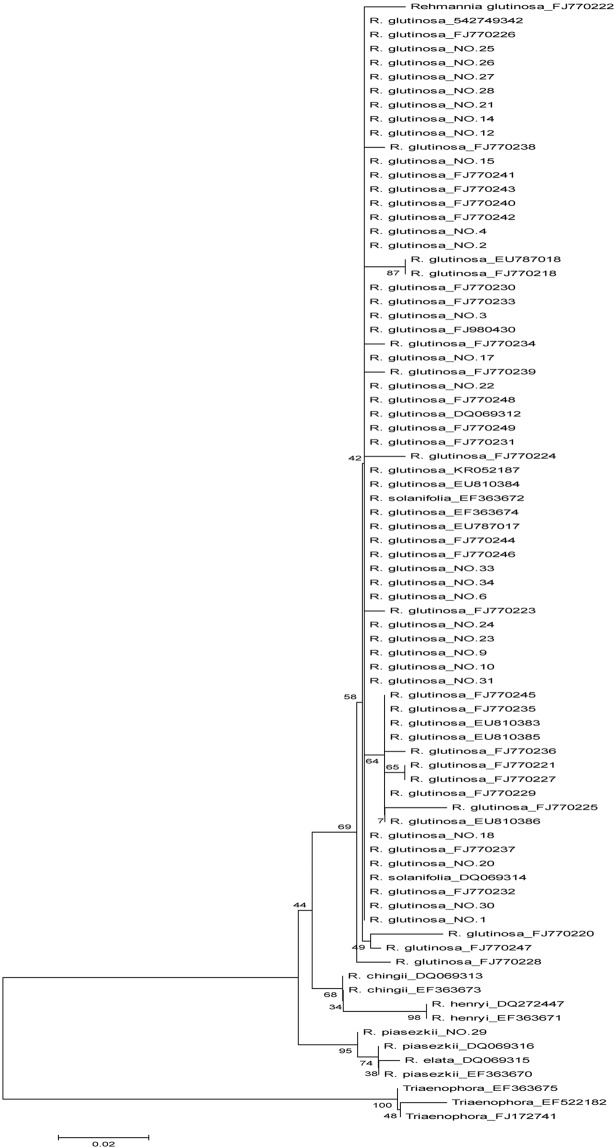
Phylogenetic tree of *Rehmannia* based on ITS2. The bootstrap scores (1000 replicates) were shown (≥50%) for each branch.

**Figure 3:**
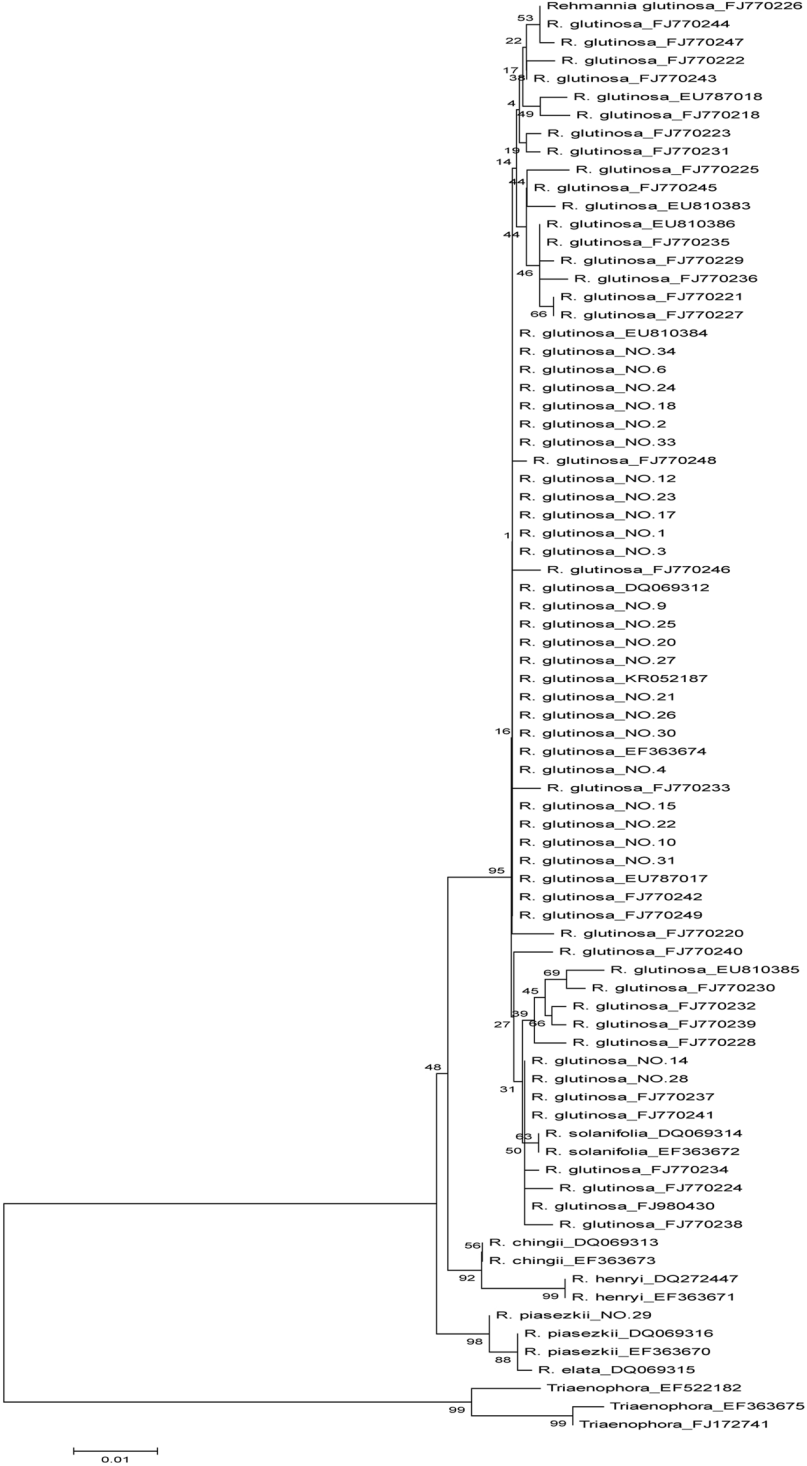
Phylogenetic tree of *Rehmannia* based on ITS. The bootstrap scores (1000 replicates) were shown (≥50%) for each branch.

In order to further evaluate these candidate barcodes in *Rehmannia*, the molecular phylogenetic tree of *Rehmannia* and its related genera *Triaenophora* was constructed using NJ method by MEGA5.0. As shown in phylogenetic tree constructed with ITS2 (Fig. 2), *Rehmannia* species were grouped into cluster I and discriminated from *Triaenophora*, cluster I was composed of two subclusters. In subcluster I, *R. glutinosa* and *R. solanifolia* were clustered together, *R. chingii* and *R. henryi* had obvious monophyley, and could be discriminated from each other. In subcluster II, *R. piasekii* and *R*. *elata* were clustered, and got 95% support rate. Similarly, in phylogenetic tree based on ITS (Fig. 3), *Rehmannia* could be discriminated from *Triaenophora*, and was divided into two subclusters. In subcluster I, cultivars and wild varieties of *R. glutinosa* and *R. piasezkii* were clustered together with 95% support rate, *R. chingii* and *R. henryi* were clustered with 92% support rate, while *R. piasekii* and *R*. *elata* were clustered in subcluster II with 98% support rate (Fig. 3).

**Figure 4:**
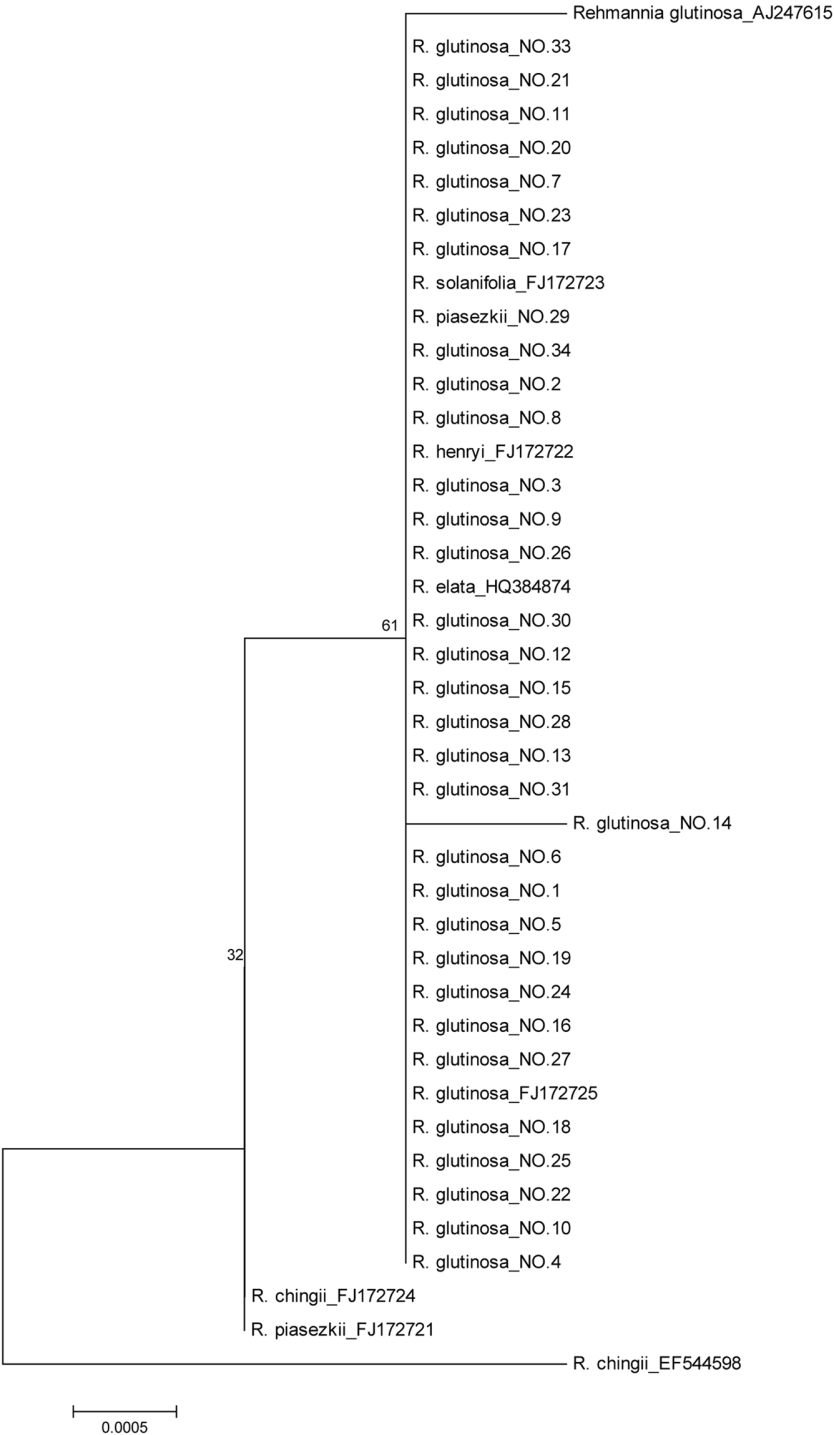
Phylogenetic tree of *Rehmannia* based on *rbcL*. The bootstrap scores (1000 replicates) were shown (≥50%) for each branch.

As shown in Fig. 4, phylogenetic tree based on *rbcL* indicated *Rehmannia* species were divided into two clusters, cluster I was composed of two subclusters. *R. glutinosa*, *R. solanifolia*, *R. piasezkii, R. henryi* and *R. elata* were clustered together in subcluster I, subcluster II was composed of *R. piasezkii* and *R. chingii*, but *R. chingii* was also found in Cluster II (Fig. 4), suggesting *Rehmannia* species could not be distinguished with *rbcL*, similar result was also found in phylogenetic tree based on *matK* (Fig. S6). In addition, phylogenetic tree based on *psbA-trnH* indicated that *Rehmannia* species were divided into two clusters, in cluster I, cultivars or wild varieties of *R. glutinosa* were respectively clustered together, while *R. piasezkii* was alone in cluster II (Fig. S7). Similarly, in phylogenetic tree based on ITS2 + *psbA-trnH*, cultivars or wild varieties of *R. glutinosa* were respectively clustered together, and were clearly separated from *R. piasezkii* (Fig. S8).

## Discussions

### The amplification of candidate barcode

As reported in some studies^16–18^, ITS, ITS2 and *psbA-trnH* could be amplified with their universe primers in *Rehmannia*, however amplification primers of *rbcL* and *matK* need to be screened. The full-length sequence of *rbcL* was amplified in *Rehmannia* by the amplification of two overlapping segments^19,20^, four pairs of *rbcL* primers 1F-724R, 636F-1368R^19^, 5′F-z895R, z674F-3′R^21^ were used, these primers had better amplification effects, especially primers 1F-724R and 636F-1368R which were more suitable to the characteristics of DNA barcode universal primers^22^. At present, the universality of *matK* primers has been controversial^9,23,24^. In this study, the full-length sequence of *matK* was successfully amplified in *Rehmannia* by primers trnk3914F-trnk2R or 1F-trnk2R, but their length was different, 2500 bp and 1800 bp, respectively, and the amplification efficiency of primer 1F-trnk2R was higher, thus the suitable primers for full-length amplification of *matK* were 1F-trnk2R in *Rehmannia*.

Compared with other candidate barcodes in *Rehmannia*, overlapping peaks were found in some sequencing atlas of ITS, and there were poly structures or repeat sequences in ITS, indicating that ITS was difficultly sequenced, so ITS sequence was re-sequenced and proofread with ITS2 sequence of *Rehmannia*, which was also found in other research^25^. Considering that mutation rate of ITS was higher^25^, plant working group of Chinese DNA barcode recommend ITS/ITS2 as core barcode for plant, ITS2 would effectively compensate when ITS is difficultly amplified and sequenced^2^.

### Feature analysis of candidate barcode

DNA barcode must have sequence variation, conserved flanking loci^26^, and short target DNA region^22^. Compared with other candidate barcodes in *Rehmannia*, the variation degree of ITS2 was the highest in *Rehmannia*, while was lower in *rbcL* and *matK*, furthermore, ITS and ITS2 had more informative sites. As a whole, ITS and ITS2 in *Rehmannia* had some characteristics of DNA barcode, such as higher aberration rate, more informative sites, shorter sequence length and better primer universality, which would be helpful to reconstruct phylogenetic relationship and identify species in *Rehmannia*.

Sequence alignment showed that *rbcL* in *Rehmannia* was highly conserved with 99.98% interspecific similarity, and not suitable for the identification of *Rehmannia* species, which was also found in *Dendrobium*^27^, Newmaster considered that *rbcL* was more suitable for the identification of plants in family, genus and above taxa^28^. However, sequence variation of *rbcL* in *Rehmannia* centrally distributed 500 bp–1000 bp, and could be amplified for the higher aberration rate of *rbcL* in *Rehmannia*. *matK* in *Rehmannia* was also highly conserved with 98.43% similarity, but the sequence of *matK* was obviously different between cultivars and wild species of *R. glutinosa*, by which *R. glutinosa* could be classified. Furthermore, *rbcL* and *matK* were successfully used to discriminate *Amana honda* or *Gentiana* from their counterfeits^29,30^. Therefore, these candidate DNA barcodes should be appropriately used for the identification of *Rehmannia* at different taxonomic level.

### Genetic divergence of candidate barcode

Genetic distances of candidate barcodes in *Rehmannia* were compared, it was found that ITS and ITS2 had higher interspecific divergence and lower intraspecific divergence in *Rehmannia*, and their minimum interspecific distance was more than coalescent depth, especially was evident in ITS2, which was similarly reported^31^. Although interspecific variation of *psbA-trnH* in *Rehmannia* was also greater than its intraspecific variation, the difference was small, Yang *et al*. found that the interspecific variation of *psbA-trnH* in *Cinnamomum cassia* was far greater than its intraspecific variation, and *Cinnamomum cassia* could be successfully identified by *psbA-trnH*^32^. Furthermore, the minimum interspecific distance of *matK* or *rbcL* in *Rehmannia* was far less than its coalesceent depth, and was not suitable for the identification of *Rehmannia* species. Thus, ITS2 and ITS might be optimal in the identification of *Rehmannia* species, which was similar in *Isatis indigotica Fort*. (*Cruciferae*)^33^.

As reported, *Rhizoma zedoariae* could be successfully identified by ITS2 + *psbA-trnH*, barcoding gap of ITS2 + *psbA-trnH* was significantly superior to *matk*, *rpoC1* and *rpoB*, and there were significant differences between their interspecific and interspecific variations^34^. In this study, the obvious barcoding gap was found in *psbA-trnH* or ITS2 + *psbA-trnH*, the overlap between interspecific and intraspecific variation of ITS, ITS2 or *matK* was less, but was more in *rbcL*. Compared with *rbcL* in *Osmunda japonica*, *psbA-trnH* had higher interspecific diversity and larger barcoding gap, was suitable for the distinguishment of *Osmunda japonica*^35^. Wilcoxon signed rank tests confirmed that the interspecific variation of ITS2 in *Rehmannia* was extremely significantly greater, and the variation degree was successively ITS2 > ITS > *psbA-trnH* > *matK > rbcL*, indicating ITS2 had obvious variability^36^.

### Identification ability of candidate barcodes

In phylogenetic tree based on ITS and ITS2 of *Rehmannia*, *R. glutinosa* and *R. solanifolia* were clustered into one branch and not be distinguished from each other, but could be separated from other *Rehmannia* species, suggesting that *R. glutinosa* and *R. solanifolia* had close relationship. Similarly, *R. elata* and *R. piasezkii* were clustered together, and also could not be distinguished from each other. Although *R. chingii* and *R. henryi* were clustered together, they could be accurately distinguished. These results were also found in other research on the relationship of *Rehmannia* based on ITS2 or ITS^1,13^, and Yan *et al*. found that *R. elata* and *R. piasezkii* might belong to the same species^13^.

In addition, Cheng *et al*. discovered that the combination of *trnS-trnG* and ITS had 100% resolution in *Rehmannia* species compared with ITS, *trnL-trnF*, *trnM-trnV* or *trnS-trnG*^14^. As shown in phylogenetic tree based on the combination of ITS2 and *psbA-trnH* or *psbA-trnH* of *Rehmannia*, cultivars and wild varieties of *R. glutinosa* were respectively clustered together, and were clearly separated from *R. piasezkii*, suggesting that cultivars and wild varieties of *R. glutinosa* could be distinguished by *psbA-trnH* or ITS2 + *psbA-trnH*. Even if the phylogenetic tree based on *matK or rbcL* indicated that they were not suitable for the identification of *Rehmannia* species, cultivars or wild varieties of *R. glutinosa* could be respectively clustered together and separated by *matK*.

### Evaluation of candidate barcodes

As is well known, ITS2 has an important significance for phylogenetic reconstruction and species classification of eukaryotic organism^37,38^. Compared with *psbA-trnH*, *matK*, *rbcL*, *rpoC1*, *ycf5* and ITS in medicinal plants, ITS2 was the most suitable for identification of medicinal plants and was recommended as universe DNA barcode of medicinal plant^10^, some research also confirmed that ITS2 could be used as universe DNA barcode of plant at different taxonomy level^39^. In this study, compared with other candidate barcodes in *Rehmannia*, ITS2 had good primer universality, was easily amplified and sequenced, and showed the highest interspecific diversity in *Rehmannia* species, which was similar to other research^40,41^. ITS of *Rehmannia* also had abundant interspecific diversity and significant interspecific divergence, and was widely applied in species identification because of its higher variability^42^. However, ITS was difficultly amplified and sequenced^2,25^, in this study, ITS of *Rehmannia* was more difficultly amplified than other candidate barcodes, and needed to be re-sequenced and proofread with ITS2 of *Rehmannia*.

Furthermore, *psbA-trnH* has good primer universality, is easily amplified and sequenced, and its interspecific variation is bigger compared with other chloroplast genes^8^, which is consistent with this experimental results. Because the evolutionary rate of *psbA-trnH* is faster, *psbA-trnH* is recommended as potential DNA barcode of plant^43^, and can be used to distinguish *Cinnamomi Cortex* from its adulterants accurately^32^. In this study, it was also found that *psbA-trnH* of *Rehmannia* had good identification ability, and its interspecific divergence was lower than that of ITS2 because of the limited number of samples and varieties. However, ITS2 + *psbA-trnH* in *Rehmannia* had higher genetic divergence and obvious barcoding gap, and had been successfully used to establish the preliminary identification system of medicinal materials^40^. Although *rbcL* was easily amplified in plants^44^, its interspecific divergence was lower among various species in the same genus, especially the closely related species^19,43^, and was not suitable for the identification at species level^10^, which was also confirmed in this study. As reported that the identification ability of *matK* in *Dendrobium* was higher compared with *rbcL*^45^, the interspecific divergence of *matK* in *Rehmannia* was also lower, and was not suitable for classification and identification of *Rehmannia* species, but could distinguish cultivars of *R. glutinosa* from its wild varieties, other research also found that *matK* can be used as standard DNA sequence for identification of *Caulis Spatholobi* and its adulterants^46^.

## In Conclusion

DNA barcoding is regarded as the global standard of species identification, but there are still debates on which DNA region can be used as the standard barcode for land plants. In this study, five candidate DNA barcodes ITS, ITS2, *matK*, *rbcL* and *psbA-trnH* were evaluated in *Rehmannia*. After primer screening and PCR amplification optimization, PCR reaction condition and universal primers of candidate barcodes were established, the rate of successful sequencing or sequence obtained was 100%, but some ITS sequences need to be proofread according to ITS2 sequences. Compared with *rbcL*, *matK* and *psbA-trnH*, ITS and ITS2 had higher mutation rate and more information sites, and ITS2 had higher interspecific diversity and lower intraspecific variation, but the interspecific genetic variation of *rbcL* and *matK* was lower. Furthermore, the obvious barcoding gap was found in *psbA-trnH* or ITS2 + *psbA-trnH*, but the overlap between interspecific and intraspecific variation of *rbcL* was more. In addition, the phylogenetic tree based on ITS or ITS2 sequence showed that *R. glutinosa*, *R. chingii* or *R. henryi* with obvious monophyly could be successfully identified, but *R. piasezkii and R. elata* were clustered into one branch, *R. solanifolia* could not be distinguished from *R. glutinosa*, and *R. chingii* was closer to *R. henryi*. In phylogenetic tree based on *psbA-trnH* or ITS2 + *psbA-trnH*, cultivars and wild varieties of *R. glutinosa* could be distinguished, were clearly separated from other species in *Rehmannia*, and cultivars or wild varieties of *R. glutinosa* could be also distinguished by *matK*. Thus, ITS2 has great potential in systematic study and species identification of *Rehmannia*, ITS2 + *psb*A-*trn*H had practical significance in the classification and identification of *Rehmannia* species, and might be the most suitable DNA barcode for *Rehmannia* species, which would provide reference data for screening of DNA barcode and species discrimination in plant, furthermore could provide theory basis for the identification of herbal medicine from medicinal plant.

## Materials and Methods

### Plant materials

In this study, experimental materials include fresh plants from different cities and counties, P. R. China (Table 1), and related sequences of *Rehmannia* species from GenBank (Table 2). All experimental samples were tentatively identified to species based on morphological characteristics by professional botanists.

### DNA extraction and detection

Total genomic DNA of experimental samples were extracted with CTAB protocol, and detected by agrose gel electrophoresis and ultraviolet spectrophotometer^47^.

### PCR amplification and sequencing

Amplification primers of target sequences in *Rehmannia* were designed according to the appropriate region of candidate barcodes (Table S5), and target sequences were amplified by PCR according to amplification conditions in Table S6. PCR amplification volume was 25 μl, and composed of 12.5 μl reaction buffer (2xTaq Master Mix) (Vazyme, Nanjing, P. R. China), 0.5 μl each primer (10 mM), 1 μl template DNA and 10.5 μl ddH_2_O. Sequencing of target sequences was performed by GENEWIZ. Inc. (Suzhou, P. R. China), DNA sequences were all submitted to GenBank (Table 1).

### Sequence splicing and correction

In order to delete primer sequence and low qualitative segments at two ends, sequence splicing and correction from sequencing atlas were performed with DNASTAR7.0 and CodonCode Aligner 5.1.5. Redundant sequences of 5.8S and 28S were removed from ITS2 sequences based on HMMer for their motifs and predictive secondary structures^38^, redundant sequences of *psbA* and *trnH* were removed from *psbA-trnH* sequences, 18S and 26S were removed from ITS sequences based on their annonation in GenBank, redundant fragments of *mat*K were removed, and low qualitative segments at two ends of *rbc*L were also removed.

### Data analysis

These candidate DNA barcodes were aligned by BLAST in GenBank and were analyzed by Clustal X 2.1 for multiple sequence alignment. Genetic distance was computed with Kimura two-parameter (K2P) model of MEGA 5.0^48^, barcoding gap was detected as reported by Meyer^31^, and wilcoxon signed rank tests were done by SPSS17.0. The phylogenetic trees were constructed using NJ method by MEGA 5.0, and bootstrap testing of 1000 replicates was performed^48^.

## Supplementary information

Supplementary materials
