# Supplementary material for: The screening and identification of DNA barcode sequences for Rehmannia
Source: Sci Rep. 2019 Nov 21;9:17295. doi: 10.1038/s41598-019-53752-8 (PMC6872558; doi:10.1038/s41598-019-53752-8)
Supplement: Supplementary file 1 — Supplementary materials [file 41598_2019_53752_MOESM1_ESM.pdf]

The screening and identification of DNA barcode sequences for *Rehmannia*

Hongying Duan, Wanshen Wang, Yunpeng Zeng, Mengmeng Guo, Yanqing Zhou*

Supplementary information

Fig.S1 PCR amplification results of ITS in *Rehmannia*

Fig.S2 PCR amplification results of ITS2 in *Rehmannia*

Fig.S3 PCR amplification results of *rbcL* in *Rehmannia*

Fig.S4 PCR amplification results of *matK* in *Rehmannia*

Fig.S5 PCR amplification results of *psbA-trnH* in *Rehmannia*

Fig.S6 Phylogenetic tree of *Rehmannia* based on *matK*

The bootstrap scores (1000 replicates) were shown (≥50%) for each branch.

Fig.S7 Phylogenetic tree of *Rehmannia* based on *psbA-trnH*

The bootstrap scores (1000 replicates) were shown (≥50%) for each branch.

Fig.S8 Phylogenetic tree of *Rehmannia* based on ITS2+*psbA-trnH*

The bootstrap scores (1000 replicates) were shown (≥50%) for each branch.

Table S1 The efficiency of PCR amplification for candidate barcodes

Table S2 The similarity comparison of candidate barcodes in *Rehmannia* by BLAST

Table S3 Wilcoxon signed rank test for interspecific variations between different sequences

Table S4 Wilcoxon signed rank test for intraspecific variations between different sequences

Table S5 Primers used in this study

Table S6 PCR reaction conditions used in this study

Supplementary information

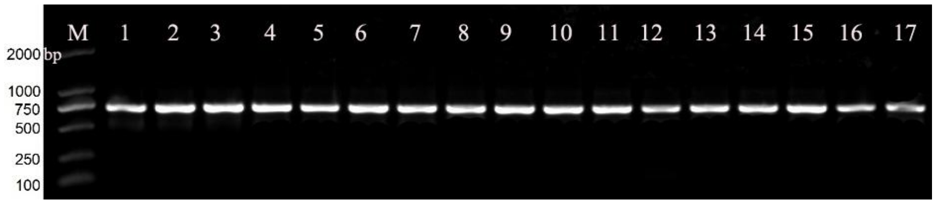

Fig.S1 PCR amplification results of ITS in *Rehmannia*

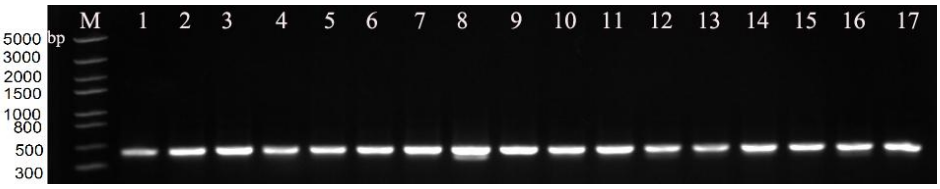

Fig.S2 PCR amplification results of ITS2 in *Rehmannia*

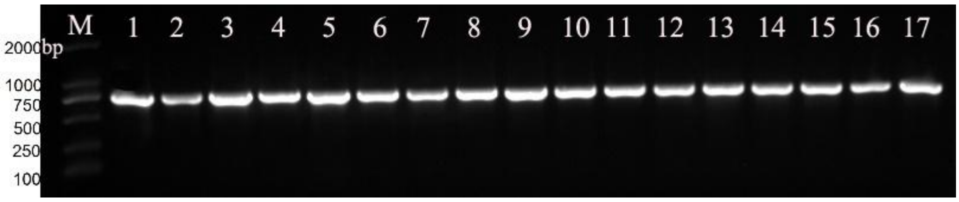

Fig.S3 PCR amplification results of *rbcL* in *Rehmannia*

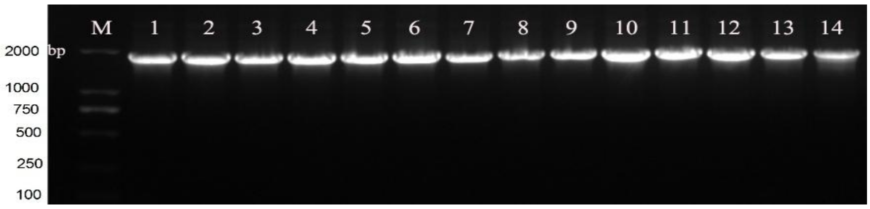

Fig.S4 PCR amplification results of *matK* in *Rehmannia*

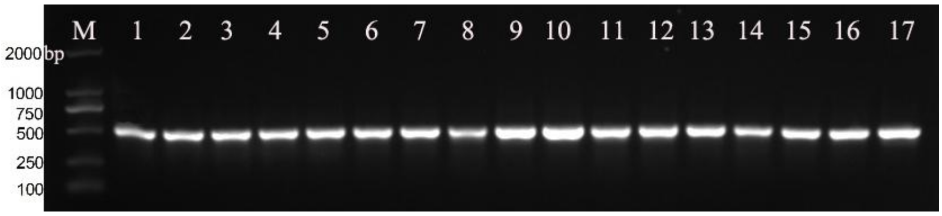

Fig.S5 PCR amplification results of *psbA-trnH* in *Rehmannia*

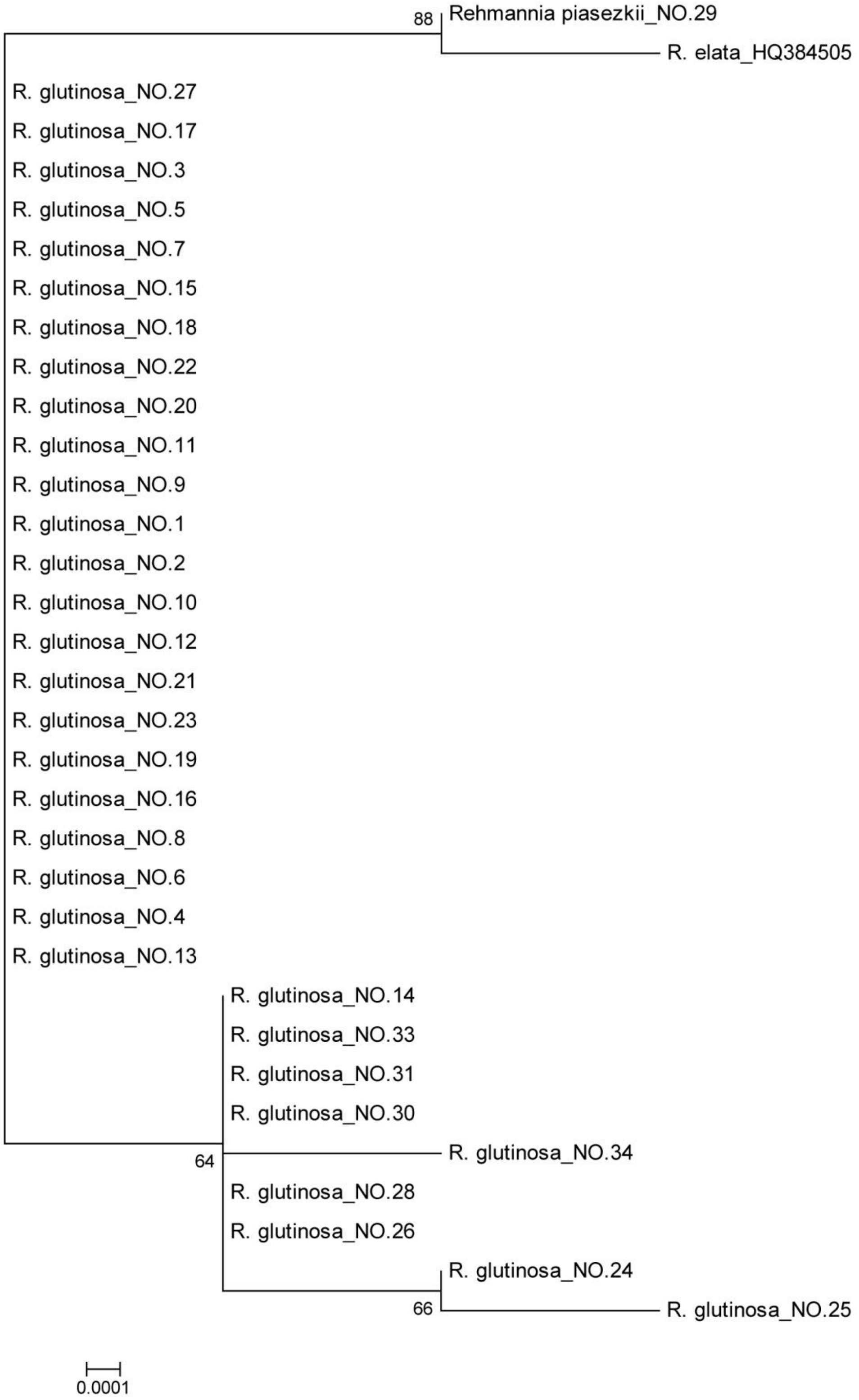

Fig.S6 Phylogenetic tree of *Rehmannia* based on *matK*

The bootstrap scores (1000 replicates) were shown (≥50%) for each branch.

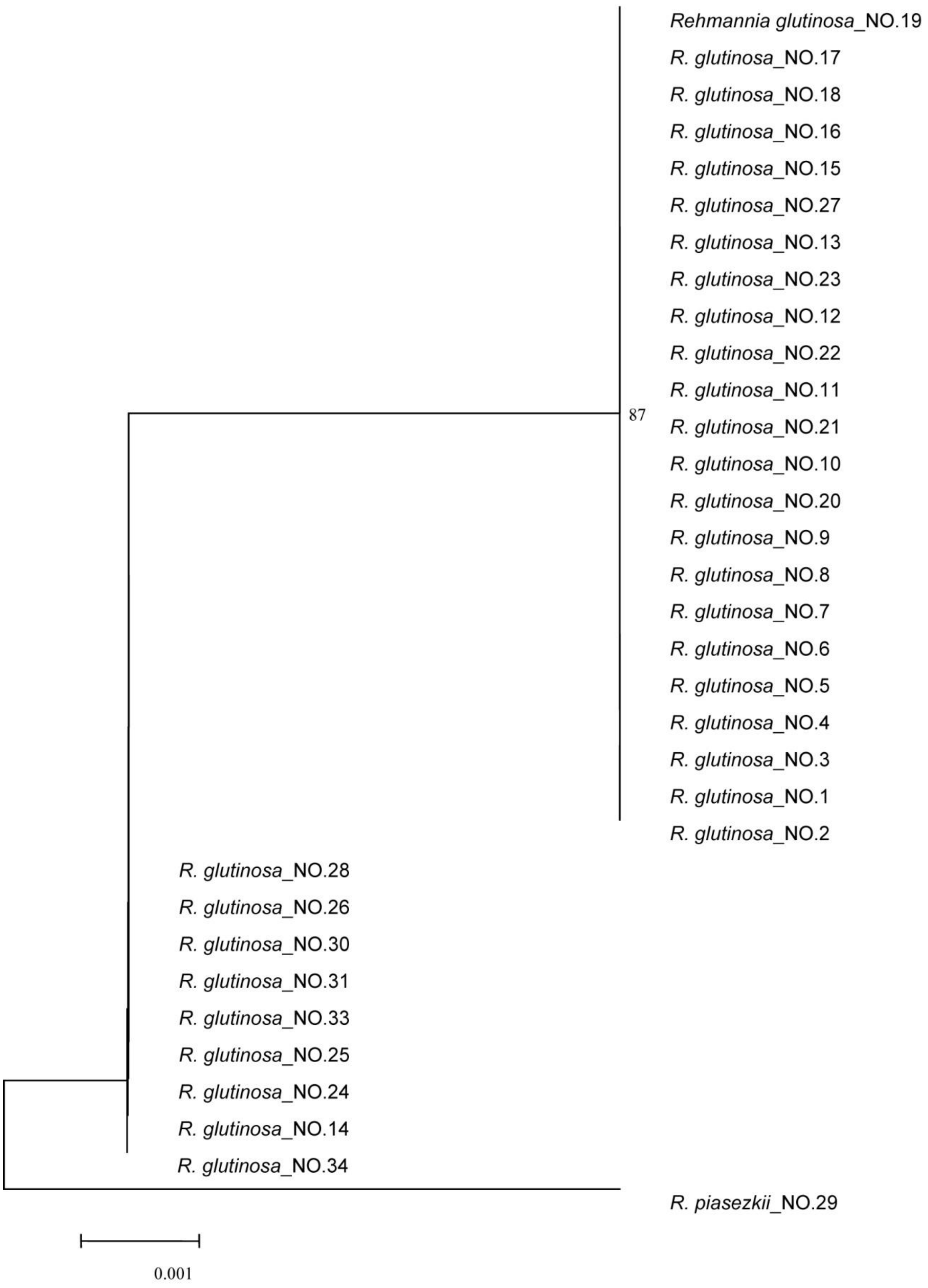

Fig.S7 Phylogenetic tree of *Rehmannia* based on *psbA-trnH*

The bootstrap scores (1000 replicates) were shown (≥50%) for each branch.

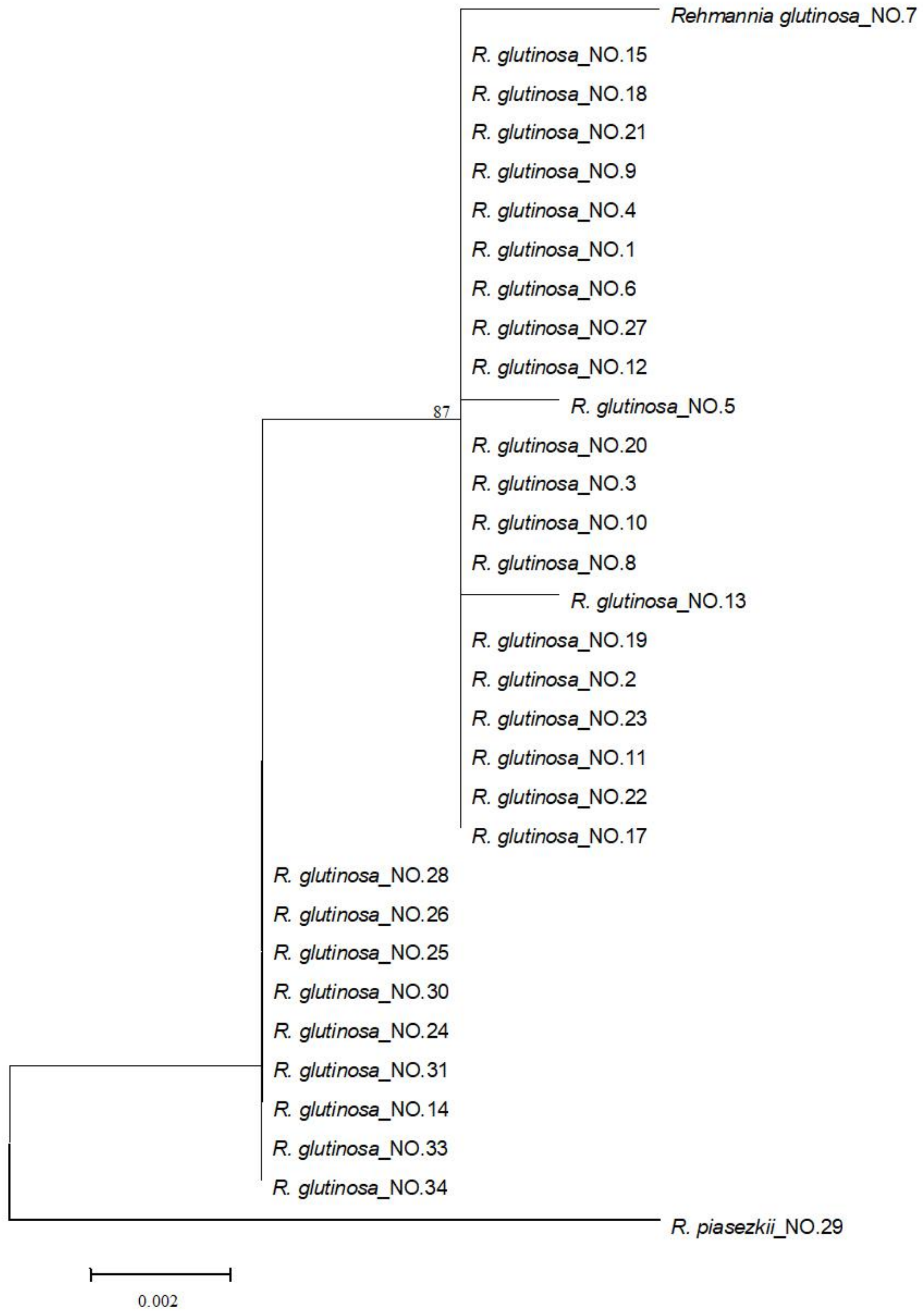

Fig.S8 Phylogenetic tree of *Rehmannia* based on ITS2+*psbA-trnH*

The bootstrap scores (1000 replicates) were shown (≥50%) for each branch.

Table S1 The efficiency of PCR amplification for candidate barcodes

Marker	Amplification rate	Sequencing rate	Sequences obtained rate
ITS	100	100	100
ITS2	100	100	100
rbcL	100	100	100
matK	100	100	100
psbA-trnH	100	100	100

Table S2 The similarity comparison of candidate barcodes in *Rehmannia* by BLAST

Marker	NO.	Species	ID	Identity%
ITS	1	*R. glutinosa* cultivar Dihuang 85-5	EU787017	100
		R. glutinosa voucher XZ-2004-04-005	EF363674	100
		R. glutinosa voucher HAAS-DH85-5	EU810384	100
	14/28	*R. glutinosa* voucher PS1518MT01	FJ980430	100
		R. glutinosa cultivar No.1 Shennongshan	FJ770237	100
		R. glutinosa voucher XZ-2004-04-005	EF363674	99
	29	*R. piasezkii* voucher XZ-2004-04-001	EF363670	99
		R. piasezkii	DQ069316	99
matK		*R. elata*	HQ384505	98
rbcL		*R. chingii*	EF544598	99
		R. glutinosa	AJ247615	99
		R. elata	HQ384874	100
		R. henryi	FJ172722	100
		R. piasezkii	FJ172721	99
		R. glutinosa	FJ172725	100

Table S3 Wilcoxon signed rank test for interspecific variations between different sequences

W^{+}	W^{-}	Inter relative ranks，n，*P* value	Result
ITS2	ITS	W^{+}=231533，W^{-}=11720，n=699，*P*=0.000	ITS2>ITS
ITS	*psbA-trnH*	W^{+}=496，W^{-}=0，n=31，*P*=0.000	ITS>*psbA-trnH*
psbA-trnH	*matK*	W^{+}=528，W^{-}=0，n=32，*P*=0.000	*psbA-trnH*>*matK*
rbcL	*matK*	W^{+}=0，W^{-}=2145，n=65，*P*=0.000	*rbcL*<*matK*

Table S4 Wilcoxon signed rank test for intraspecific variations between different sequences

W^+	W^-	Inter relative ranks，n，*P* value	Result
ITS2	ITS	W^+=638743，W^-=807107，n=2151，*P*=0.000	ITS2<ITS
ITS	*psbA-trnH*	W^+=25273.5，W^-=27052.5，n=465，*P*=0.593	ITS=*psbA-trnH*
matK	*psbA-trnH*	W^+=242.5，W^-=25863.5，n=496，*P*=0.000	*matK*<*psbA-trnH*
rbcL	*matK*	W^+=460，W^-=21068，n=496，*P*=0.000	*rbcL*< *matK*

Table S5 Primers used in this study

Marker	Primer	Direction	Sequence 5’→3’
ITS	T5	F	GGAAGTAAAAGTCGTAACAAGG
	T4	R	TCCTCCGCTTATTGATATGC
ITS2	T3	F	GCATCGATGAAGAACGCAGC
	T4	R	TCCTCCGCTTATTGATATGC
rbcL	1F	F	ATGTCACCACAAACAGAAAC
	724R	R	TCGCATGTACCTGCAGTAGC
	636F	F	GCGTTGGAGAGATCGTTTCT
	1368R	R	CTTTCCAAATTTCACAAGCAGCA
rbcL	5’F	F	ATGTCACCACAAACAGAAACTAAAGC
	z895R	R	ACCATGATTCTTCTGCCTATCAATAACTGC
	z674F	F	TTTATAAATCACAAGCCGAAACTGGTGAAATC
	3’R	R	CTTTTAGTAAAAGATTGGGCCGAG
matK	1F	F	ACTGTATCGCACTATGTATCA
	trnk2R	R	AACTAGTCGGATGGAGTAG
	trnk3914F	F	GGGGTTGCTAACTCAACGG
psbA-trnH	trnHf_05	F	CGCGCATGGTGGATTCACAATCC
	psbA 3’f	R	GTTATGCATGAACGTAATGCTC

Table S6 PCR reaction conditions used in this study

Marker	Primer	Condition
ITS	T5	94℃ 3min，30cycles（94℃ 30s，46℃ 30s，72℃ 60s），72℃
	T4	10min
ITS2	T3	94℃ 3min，30cycles（94℃ 30s，48℃ 30s，72℃ 60s），72℃
	T4	10min
rbcL	5’F	94℃ 4min，30cycles（94℃ 30s，60℃ 30s，72℃ 60s），72℃
	z895R	10min
	z674F	94℃ 4min，30cycles（94℃ 30s，52℃ 30s，72℃ 60s），72℃
	3’R	10min
rbcL	1F	94℃ 3min，30cycles（94℃ 30s，50℃ 30s，72℃ 1min），
	724R	72℃ 10min
	636f	94℃ 3min，30cycles（94℃ 30s，50℃ 30s，72℃ 1min），
	1368r	72℃ 10min
matK	1F	94℃ 5min，30cycles（94℃ 90s，48.6℃ 2min，72℃ 1min），
	trnk 2R	72℃ 15min
	trnk 3914F	94℃ 4min，35cycles（94℃ 30s，48℃ 1min，72℃ 2min），
	trnk 2R	72℃ 10min
psbA-trnH	psbA 3’f	94℃ 4min，35cycles（94℃ 30s，55℃ 30s，72℃ 1min），
	trnHf_05	72℃ 10min
